# Genome Organization and Copy-Number Variation Reveal Clues to Virulence Evolution in *Coccidioides posadasii*

**DOI:** 10.3390/jof8121235

**Published:** 2022-11-22

**Authors:** Claire A. Dubin, Mark Voorhies, Anita Sil, Marcus M. Teixeira, Bridget M. Barker, Rachel B. Brem

**Affiliations:** 1 Department of Plant and Microbial Biology, UC Berkeley, Berkeley, CA 94720-3102, USA; 2 Department of Microbiology and Immunology, UC San Francisco, San Francisco, CA 94143, USA; 3 The Translational Genomics Research Institute (TGen)-Affiliate of City of Hope, Flagstaff, AZ 85004, USA; 4 Pathogen and Microbiome Institute, Northern Arizona University, Flagstaff, AZ 86011, USA; 5 Núcleo de Medicina Tropical, Faculdade de Medicina, Universidade de Brasília, Brasília 70910-900, Brazil

**Keywords:** *Coccidioides*, virulence, fungal pathogens, copy number variation, genome organization

## Abstract

The human fungal pathogen *Coccidioides* spp. causes valley fever, a treatment-refractory and sometimes deadly disease prevalent in arid regions of the western hemisphere. Fungal virulence in the mammalian host hinges on a switch between growth as hyphae and as large spherules containing infectious spores. How these virulence programs are encoded in the genome remains poorly understood. Drawing on *Coccidioides* genomic resources, we first discovered a new facet of genome organization in this system: spherule-gene islands, clusters of genes physically linked in the genome that exhibited specific mRNA induction in the spherule phase. Next, we surveyed copy-number variation genome-wide among strains of *C. posadasii*. Emerging from this catalog were spherule-gene islands with striking presence–absence differentiation between *C. posadasii* populations, a pattern expected from virulence factors subjected to different selective pressures across habitats. Finally, analyzing single-nucleotide differences across *C. posadasii* strains, we identified signatures of natural selection in spherule-expressed genes. Together, our data establish spherule-gene islands as candidate determinants of virulence and targets of selection in *Coccidioides*.

## 1. Introduction

*Coccidioides* spp. are fungal pathogens endemic to desert regions in the Western Hemisphere. *Coccidioides* infects, colonizes, and can kill immunocompetent individuals when they inhale spores from soils, with a high risk among agricultural and construction workers in endemic areas [1,2]. The ability of *Coccidioides* to cause disease depends on an elaborate developmental transition from hyphae to airborne spores called arthroconidia, and then to a host form known as spherules. The latter fill with vegetative endospores and ultimately rupture, enabling dissemination to new infection sites within the host [3].

Against a backdrop of candidate-gene studies of *Coccidioides* virulence factors [4,5], the molecular mechanisms of *Coccidioides* virulence remain poorly understood. However, genome [6,7,8,9,10] and transcriptome [11,12,13,14] data in this organism raise the possibility of incisive in silico analyses of candidate virulence genes. In other fungal pathogens, as a complement to genomic screens [15,16], the study of genome architecture has proven to accelerate the discovery of virulence genes—particularly when they group into virulence islands [17,18,19,20]. By the same token, population genomics has proven to be a powerful approach in other fungi for the discovery of how and why virulence factors diversify across isolates in the wild. Indeed, rapid evolution is an identifying characteristic of the genes of fungal virulence programs [17,18,19,20,21]. 

We set out to use computational genomic strategies to investigate the organization, function, and evolution of candidate virulence factors in *Coccidioides*. Our results revealed evidence for virulence island-like loci in this fungus, including secondary metabolite clusters expressed in spherules, and we documented accelerated evolution of virulence-relevant genes in wild *Coccidioides*, as well as widespread copy number variation. 

## 2. Methods

### 2.1. Sequence and Population Information

The *C. posadasii* strain Silveira’s reference genome sequence and annotation data were downloaded from Genbank, accession numbers CP075068–CP075072 [9]. PFAM domain, secondary metabolite, signal peptide, carbohydrate active enzyme, and transmembrane domain annotations were extracted from these files. *C. posadasii* transposable element sequences were downloaded [22] and used as input into RepeatMasker [23] to annotate transposable elements and repetitive regions in the *C. posadasii* genome. *C. immitis* and *Uncinocarpus reesii* homologs to genes in the chromosome III copy number variable region were found using Protein BLAST [24].

*C. posadasii* isolates were assigned to populations using assignments from [10,25]. Isolates with evidence of admixture or inconsistent population classifications across studies were not included in population-level analyses. After these filters, 29, 8, and 4 strains in the Arizona, Texas/Mexico/South America, and Caribbean populations, respectively, were retained for further analyses (Table S1). Short-read FASTQ files for isolates were downloaded from the European Nucleotide Archive, project PRJNA274372. We used BWA-MEM [26] to align reads from each strain to the *C. posadasii* strain Silveira reference genome. The BWA-MEM output BAM files were used as input into Pilon version 1.23 with the variant flag [27] and exported in Variant Call Format (VCF). We removed ambiguous sites annotated as “Amb,” low coverage sites annotated as “LowCov,” and sites with coverage greater than three times the average coverage for the sample. We then combined VCFs from each strain into a multisample VCF using bcftools merge [28], then removed non-variant sites and sites with missing data in 5% or more strains using vcftools [29]. We measured pairwise genotype concordance with bcftools gtcheck [28]. For any case in which two isolates were highly similar (fewer than 250 single nucleotide polymorphism (SNP) differences), the isolate with higher average read depth was retained for analysis (Table S1). Approximately 330,000 SNPs and 50,000 insertions and deletions were used for downstream analyses.

We used the bcftools consensus to incorporate variants from each strain into the *C. posadasii* strain Silveira reference coding sequence (CDS) for all genes. Nucleotide sequences were translated into amino acid sequences using Biopython, and each sequence type was aligned using MUSCLE [30] with –maxiters 2. Codon alignments for P_N_/P_S_ calculations (see below) were generated using PAL2NAL [31]. Sequences with overlapping copy number variants (CNVs), as well as those containing premature stop codons or more than 5% gaps, were removed from the analysis. Genes for which sequences from less than 75% of the population were present were removed, leaving a pool of 7870 genes.

### 2.2. Spherule- and Hyphae-Gene Islands

Expression data for wild-type *C. posadasii* were downloaded from [12], which identified 1082 genes significantly upregulated in the spherule phase, 1200 genes significantly upregulated in the hyphae phase, and 5019 genes which were not differentially expressed across life stages. To analyze these expression patterns in inferred secondary metabolite clusters, antiSMASH (antibiotics and Secondary Metabolite Analysis Shell) annotations were retrieved from the GenBank annotations described above and integrated with differential expression calls.

In order to identify spherule- and hyphae-gene islands (genome regions enriched for genes upregulated in the respective life stage), we carried out a permutation analysis using the total number of genes induced in the stage, *n_true_*, as follows. We first divided the genome into 25 kb windows and tabulated the number of genes induced in the stage in each window. We call *w_t, true_* the number of windows encompassing at least *t* genes induced in the stage. Next, we generated a permuted localization data set, in which we randomly labeled genes across the genome as upregulated or not upregulated in the focal life stage, with a total of *n_true_* genes assigned to the former category. Using this permuted genome, we tabulated *w_t, perm_*, the number of windows which overlapped at least *t* genes induced in the stage. We repeated this calculation for 10,000 permuted genomes and used as an empirical *p*-value, the fraction for which *w_t, perm_* ≥ *w_t, true_*. We then iterated through possible values of *t* and identified those enabling highly-powered discovery of islands. Our final analysis for spherule-upregulated genes used *t* = 4 genes per window; at this value, the number of spherule-gene islands was significant at empirical *p* = 0.000145 (Supplementary Table S2). Our final analysis for hyphae-upregulated genes used *t* = 6 genes per window, yielding hyphae-gene islands which were significant at empirical *p* = 0.00715 (Supplementary Table S2).

### 2.3. Population-Genetic Statistics

For each gene of the *C. posadasii* genome, nucleotide diversity (π) was calculated using the DendroPy 4.5.2 popgenstat module [32]. Interpopulation nucleotide diversity (*D_XY_*) between the Arizona population, on the one hand, and the Texas/Mexico/South American population on the other, was calculated as

$$D_{XY}=\frac{1}{n_{AZ}n_{TXMXSA}}\overset{n_{AZ}}{\underset{i=1}{\sum }}\overset{n_{TXMXSA}}{\underset{i=1}{\sum }}d_{ij}$$

where *n* is the number of sequences from each population, and *d_ij_* is the number of sites with nucleotide differences at the same position in each pair of sequences. Per-gene measurements are shown in Supplementary Table S3, though it should be noted that we assessed significance for gene cohorts based on resampling tests (see below).

For *P_N_/P_S_*, for each gene of the genome, EggLib 3.0.0’s coding diversity module [33] was used to count the number of possible nonsynonymous polymorphic sites (*N_sites_*), possible synonymous polymorphic sites (*S_sites_*), nonsynonymous changes (*N*), and synonymous changes (*S*). *P_N_/P_S_* was then calculated as

PnPs=NNsitesSSsites

Per-gene *P_N_/P_S_* data are shown in Supplementary Table S3; we assessed the significance of this statistic across gene cohorts via resampling tests (see below).

*F_ST_*, Tajima’s D, and π were calculated between groups of strains for each 250 base pair window in the genome using vcftools 2.7.5 [29]. Per-window values are listed in Supplementary Table S4; significance evaluation used resampling tests in gene groups (see below).

To measure enrichment of nucleotide diversity in spherule-upregulated genes, we calculated *M_spherule_*, the median value of π across spherule-upregulated genes. We then sampled 10,000 random groups of genes, each the same size as the spherule-upregulated gene cohort, and calculated *M_random_*, the median value of π in each sample. We used as an empirical *p* value the proportion of samples in which *M_random_* ≥ *M_spherule_*. Enrichment tests for other statistics were calculated using an analogous approach. We note that, where appropriate, the resampling strategy controls for differences in sample size and demography between populations.

### 2.4. Copy Number Variation

To identify copy-number-variable regions in each strain, we used the BAM files generated with BWA-MEM as input into Control-FREEC [34] with the following parameters: window = 250, telocentromeric = 0, minExpectedGC = 0.33, maxExpectedGC = 0.63. This returned, for each strain, the predicted copy number of each 250 bp window of the genome.

For each 250 bp window in the output that did not overlap a transposon, we calculated *V_ST_* as:

$$V_{ST}=\frac{Var_{total}-\left[(Var_{AZ}\times N_{AZ}\right)+(Var_{TXMXSA}\times N_{TXMXSA})+(Var_{CB}\times N_{CB})]}{Var_{total}}$$

where *Var* signifies the variance of copy number in a given population and *N* signifies the number of isolates in a given population [35]. Per-window measurements across the genome are shown in Supplementary Table S5B.

The degree of enrichment for high *V_ST_* in the chromosome III region was calculated as follows. We carried out a simulation, taking into account three parameters: the total number of windows of the genome containing CNVs that had *V_ST_* above the 99th percentile, 63; the subset of these in the chromosome III region, which numbered 35; and the size of the chromosome III region, 190-kb. We simulated a genome in which, across all CNV-containing windows of the genome, we randomly marked 68 as top-scoring with respect to *V_ST_*. We then used this simulated genome to tabulate the number *n* of 190-kb regions containing at least 35 simulated top-scoring loci. We repeated this calculation 10,000 times, and we took, as an empirical *p*-value, the proportion of such simulated data sets in which *n* > 0.

## 3. Results

### 3.1. Secondary-Metabolite Clusters Induced during Spherule Formation

**Figure 1:**
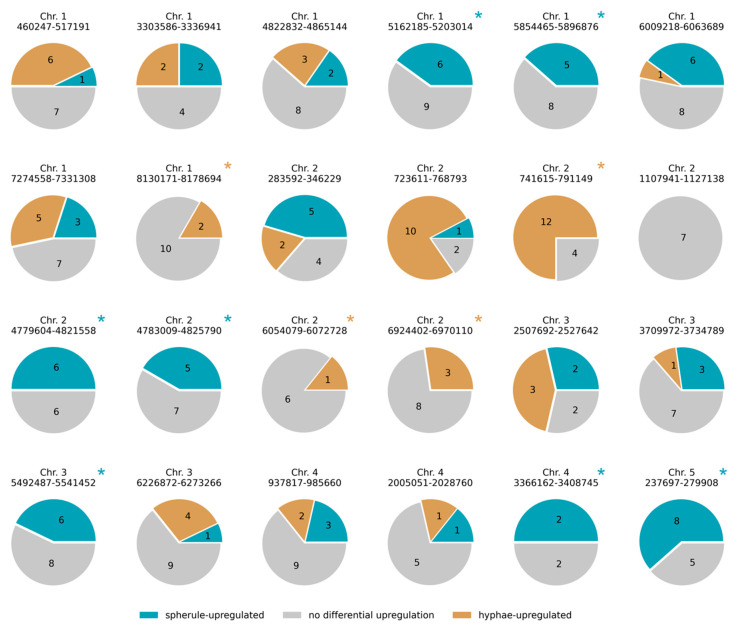
Secondary metabolite clusters with life cycle-specific upregulation. Each circle reports expression patterns from one secondary metabolite cluster. In a given circle, brightly colored areas and overlaid black numbers report the proportion and number, respectively, of genes in the cluster induced in the indicated life cycle phase of *C. posadasii*. Clusters with such expression signals in only one life cycle phase are marked with an asterisk.

In order to gain biological insight into life-stage-specific expression programs in *C. posadasii*, we made use of recent transcriptomic profiles of hyphae and spherules of this organism in standard culture conditions [12]. We first evaluated whether and how certain cohorts of genes were particularly active in each life stage. Among genes induced in hyphae, we detected an enrichment of loci encoding secreted factors and carbohydrate active enzymes (Table 1), which is expected if the fungus carries out extracellular breakdown of complex substrates when living in the soil in its hyphal form. In genes induced in spherules, we found enrichment for members of predicted secondary metabolite clusters, a trend that was robust to the source of the cluster annotations (Table 1). Inspecting individual secondary metabolite gene clusters, we identified several with predominantly hyphal-specific or spherule-specific expression (Figure 1). From these annotation data, we conclude that life-stage-specific expression programs in *C. posadasii* are functionally coherent, with particularly strong evidence for secondary metabolites in the spherule phase.

### 3.2. Rapid Nucleotide Evolution of Spherule-Expressed Genes

Trends from other fungal pathogens have made it clear that virulence genes tend to evolve rapidly relative to those of other functional classes [17,18,19,20,21]. In order to explore this notion in *C. posadasii*, we first focused on single-nucleotide differences between strains. We made use of whole-genome resequencing of clinical isolates from a well-sampled Arizona population, as well as smaller data sets from the Texas/Mexico/South America and Caribbean populations [10,25]. For each strain, we mapped raw genomic DNA reads to the *C. posadasii* reference genome (strain Silveira) [9] to call single-nucleotide variants. Resampling tests using the entire set of spherule-expressed genes (Table 2) established that these loci were particularly likely to vary in terms of nucleotide differences across Arizona strains (quantified by π, the average number of nucleotide differences between random samples of two alleles from a population) and between populations (quantified with *D_XY_,* the absolute population differentiation). These effects were of small magnitude, but robustly significant (Table 2), indicative of an uptick in evolutionary rate, on average, among genes induced in the spherule phase. An unbiased search of annotation cohorts also revealed high rates of single-nucleotide variation in phosphotransferase enzyme family genes (PF01636; Table 2), echoing previous studies on this family in other fungi [36,37].

Next, we examined protein evolutionary rate across *C. posadasii* Arizona strains. Results revealed a modest but significant enrichment for higher values in spherule-expressed genes, paralleling the trends we had seen in nucleotide changes (Table 2). We reasoned that the signal for protein variation could be attributed, in part, to diversifying the selection at individual loci. In order to explore this, we evaluated the relative proportion of amino acid polymorphism and silent changes at each gene in turn. The former were in excess in 68 spherule-expressed genes (and 414 other genes; Supplementary Table S3), each representing a signature of positive selection on the function of the respective protein. Such a given gene harbored mostly singleton amino-acid changes (Supplementary Figure S1), suggesting a history of rare variants, each accumulated independently by a lineage within Arizona, at these loci. Together, our analyses of single-nucleotide variants make it clear that spherule-expressed genes evolve rapidly across *C. posadasii* strains.

### 3.3. Identifying Spherule-Gene Islands

**Figure 2:**
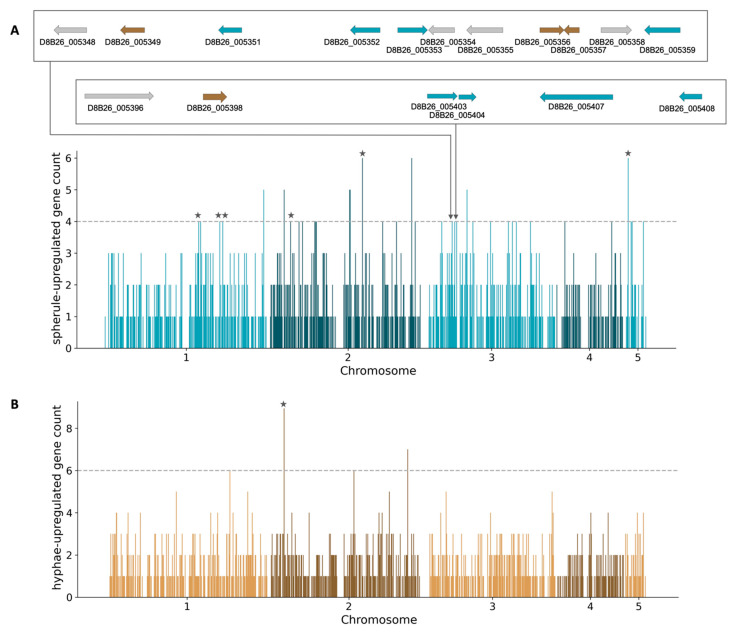
Spherule-gene and hyphae-gene islands. Each panel reports the results of a test for genomic regions over-represented among genes induced in one C. posadasii life cycle phase. The x-axis reports the chromosome positions of a given genomic window, and the y-axis reports the number of genes in the window upregulated in spherules (**A**) or hyphae (**B**). The dashed line reports the maximum number of genes of the respective induction behavior expected in a window under a null model. Stars indicate windows overlapping with secondary metabolite clusters from Figure 1, and inset tracks indicate windows in a highly variable region of chromosome III (see below).

We now aimed to search for patterns of chromosomal organization among the genes of *C. posadasii* induction programs. We developed a resampling-based test for genomic regions harboring an excess of spherule- or hyphal-expressed genes in the Silveira type strain (see Section 2). The results revealed 36 regions with a particularly high number of genes expressed in spherules, and four genomic regions in the analogous test for hyphae (Figure 2 and Supplementary Table S2). We refer to the former as spherule-gene islands. Among them, we noted six loci which we had already highlighted as secondary metabolite gene clusters with spherule induction (Figure 2). Such overlap served as validation of our unbiased approach to identify co-regulated, functionally similar genes that were physically linked, particularly in the spherule expression program. More broadly, we considered the catalog of the latter to support the inference of a physical organization of *C. posadasii* virulence genes in islands.

### 3.4. An Atlas of Copy Number Variation

Having identified spherule-gene islands in *C. posadasii*, we hypothesized that some such loci could be subject to gains and losses across strains. In order to explore this, we used whole-genome resequencing resources [10,25] as input into a pipeline to call copy number changes relative to the Silveira reference genome. 

Before focusing on spherule-gene islands (see below), we harnessed the data for a more general survey of copy number-variable regions.

**Figure 3:**
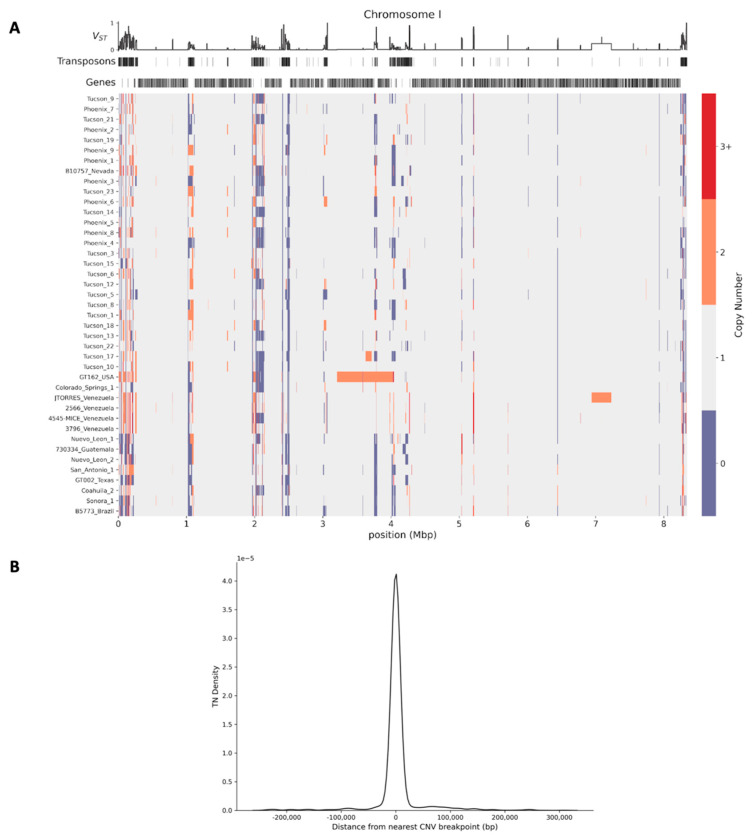
Copy number variants and their proximity to transposons across *C. posadasii* strains. (**A**) In the main panel, each row reports the results of quantification of copy number in the indicated strain relative to the C. posadasii reference assembly (Teixeira et al., 2021). The x-axis reports genome location, and the color scale reports copy number. At the top, the y-axis reports the *V_ST_* metric of population differentiation of the copy number; rows underneath report transposon and gene locations. Only results from chromosome I are shown; the remaining chromosomes are reported in Figure S2. (**B**) The x-axis reports the distance between a copy number variant (CNV) boundary and the nearest transposon; the y-axis reports the proportion of CNVs with the transposon distance on the x, as a kernel density estimate.

Most copy-number variable loci across *C. posadasii* encompassed relatively short stretches in gene-poor regions (Figure 3A, Supplementary Table S5, and Supplementary Figure S2). They tended to be flanked by transposons (Figure 3B), as expected if instability mediated by homologous transposon sequences drove their gain and loss across lineages. In most cases, the minor allele (tabulated as presence or absence relative to Silveira) manifested in only a few strains, though a handful of variants were detected at high frequency across genomes (Supplementary Figure S3).

We quantified population stratification across windows of copy-number variants using the *V_ST_* statistic [35,38] (Figure 3 and Supplementary Figure S2). Top-scoring loci in this calculation had copy number variants which were over-represented in the Arizona, Texas/Mexico/South America, or Caribbean populations; the 99th percentile of *V_ST_* genome-wide corresponded to 68 genomic windows (Supplementary Table S5). Among these *V_ST_* peaks, we noted three with gene losses unique to Texas/Mexico/South America strains (Supplementary Table S5), likely reflecting fixations during bottlenecks in the establishment of this population [10,39].

Returning to the complete set of copy number variants across *C. posadasii* strains, we sought to highlight variable loci overlapping coding genes. In total, these numbered 1157 regions (Supplementary Table S5). Two involved >100 genes each, in strain GT-162 from Texas and strain JTORRES from Venezuela, respectively (Figure 3A). Most of the remainder were smaller, with an interquartile range of ~3 kb to 19 kb (Supplementary Table S5). In order to investigate trends in function among the genes at copy number-variable sites, we focused on Arizona *C. posadasii.* Enrichment analyses of copy-number changes among Arizona strains revealed an over-representation of phosphotransferase enzyme genes (PF01636; *p* = 0.000078), paralleling the enrichment of single-nucleotide variants we had noted in this gene cohort (Table 2).

Together, these data make clear that copy-number variation is widespread in *C. posadasii*—including population-specific variants, which is suggestive of phenotypes and/or selective pressures unique to the distinct endemic areas of the fungus.

### 3.5. Widespread Deletions in a Spherule-Expressed Locus

**Figure 4:**
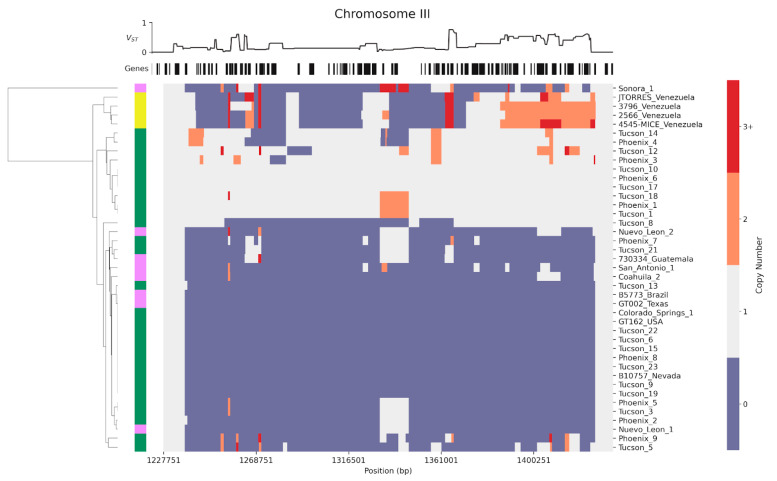
Widespread deletions in a spherule-expressed locus. In the main panel, data are as in Figure 3 and Supplementary Figure S3, except that only a region of chromosome III is shown, and strains were clustered using Euclidean distance and average linkage. For a version showing windows overlapping transposons, see Supplementary Figure S4. Cells on the left report strain population: Arizona, green; Caribbean, yellow; Texas/Mexico/South America, pink.

We aimed to investigate copy number variation in spherule-gene islands across *C. posadasii,* as a window into genomic and evolutionary forces at these sites. From the intersection between our identified sets of copy-number variant loci and spherule-gene islands, one striking case of overlap emerged (Figure 4). This was a 190-kb stretch on chromosome III, whose full extent was detectable in the genomes of only 30% of isolates from the Arizona population (grey stripe in Figure 4). Its sequence was largely undetectable in most other strains, although 3/4 Venezuelan isolates harbored a duplication of the centromeric end (rightmost orange block in Figure 4). Consequently, the whole region was an extreme outlier in terms of population differences in copy number: it encompassed 35 of the 68 genome windows scoring in the 99th percentile of *V_ST_* (top trace, Figure 4; resampling *p* < 10^−4^). Thus, *C. posadasii* populations harbor distinct haplotypes at high frequency at this chromosome III locus. Given the similarity to patterns at contingency loci [40] and sites subject to balancing selection [41,42] in other microbial pathogens, we chose to describe the genomics of the chromosome III region in more depth.

The 78 coding genes of the chromosome III region included 15 spherule-induced genes (falling into two separately identified spherule-gene islands; Figure 2) and another 9 genes induced in hyphae. This constituted a strong enrichment of spherule-induced genes across the region relative to the null expectation (Fisher’s two-sided test *p* = 0.008). Most salient among the annotated genes at this locus (Supplementary Table S6) were an inferred polysaccharide synthase for the spherule capsule; a patatin-like phosopholipase; a hemolysin; and a ferric reductase—all orthologs of known or suspected factors mediating interactions with the host in other fungal [43,44,45]. These functions established the chromosome III region as a compelling example of a putative virulence island with presence–absence variation across the species. 

**Figure 5:**
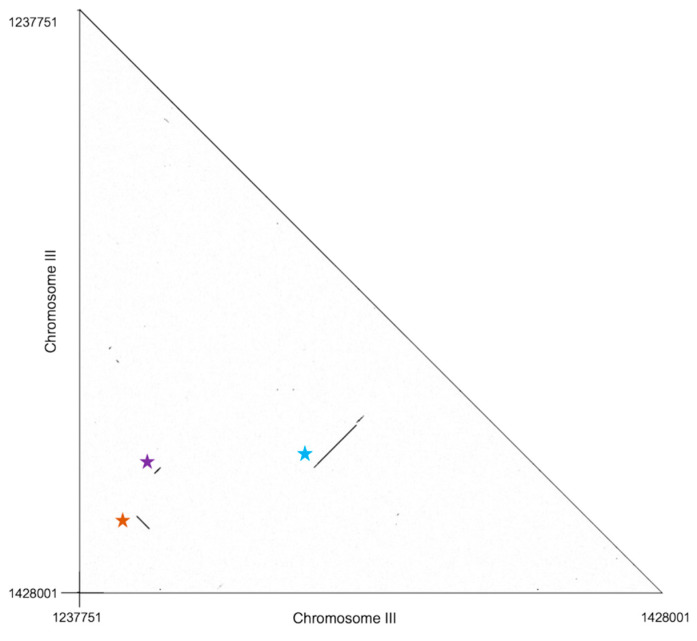
Paralogy in the copy number-variable chromosome III region. Axes report position in the *C. posadasii* Silveira reference genome in the chromosome III locus from Figure 4. Black points report nucleotide identity. Colored stars identify inferred gene duplications discussed in the Section 4.1.

Investigating the architecture of the chromosome III region, we noted extensive sequence similarity in some pairs of coding genes encompassed by the locus (Figure 5 and Section 4.1). We inferred that each such match represented a case of paralogy generated by a local duplication. This included a stretch of seven contiguous genes; a stretch of two contiguous genes; and a single duplicated gene, each present in two copies within the region in Silveira (Figure 5 and Section 4.1). Sequence identities of the gene pairs in Silveira ranged from 68% to 100% in non-gapped regions (Figure 5). Transposons were positioned throughout the chromosome III region and at its boundaries (Supplementary Figure S4), plausibly acting as drivers of rearrangements across strains.

We likewise assessed homology of each gene of the chromosome III region in *C. immitis*, the sister species to *C. posadasii*, finding *C. immitis* orthologs for most genes of the locus (Supplementary Table S6). Approximately half of the genes had homologs in a more distant relative, *U. reesii* (Supplementary Table S6). These data supported a picture in which most of the chromosome III locus existed at least as far back as the *Coccidioides* ancestor, undergoing gain and duplication of individual genes, as well as wholesale loss, within *C. posadasii*. 

We envisioned two possible models for the history of the chromosome III region in *C. posadasii*. In one scenario, a given deletion would have occurred once in a member of an ancestral *C. posadasii* population. If so, descendants of the latter, namely the extant strains we had noted that lacked the respective stretch of genome, would show elevated allele-sharing in flanking regions. Alternatively, chromosome III loss events could have occurred repeatedly in *C. posadasii* lineages, in which case extant strains with deletions would not exhibit high sequence similarity to each other in surrounding regions. In order to explore evidence for these possibilities, we used genomes from the deeply-sampled Arizona *C. posadasii* population. We categorized each Arizona strain as harboring a full deletion or full retention of the chromosome III locus. We then examined sequence variation in windows flanking the chromosome III locus. We tabulated population differentiation (using the *F_ST_* metric) between the strains of the two categories, and, separately, we calculated nucleotide diversity and Tajima’s *D*, an allele frequency metric, for each category of strains. In no case did the chromosome III locus show a signal as an outlier relative to the genomic null (Supplementary Table S4). Strains harboring the deletion also did not cluster together in the species-wide phylogeny (Supplementary Figure S5). We thus detected no allele-sharing among Arizona *C. posadasii* strains with the chromosome III deletion, supporting an inference of independent de novo events as the origin of the high frequency of the deletion haplotype.

In summary, our observations of the chromosome III locus revealed that *C. posadasii* populations have repeatedly acquired new haplotypes at this site and maintained them at high frequency, and that the region is rich with putative virulence genes. These results underscore the power of our genomic surveys to uncover candidate virulence determinants in *C. posadasii* and to track their evolution across the species.

## 4. Discussion

In this work, we have studied functional, genomic, and evolutionary correlates of the spherule expression program in *C. posadasii*. We have shown that spherule genes tend to fall into virulence-island-like regions, evolve rapidly, and participate in secondary-metabolite enzymatic pathways. We have also characterized the complex evolutionary changes at one spherule-gene island, including gains and losses of genes with functions likely relevant for interactions with the mammalian host. Our genomic data serve as a hypothesis-generating framework for virulence determinants in this under-studied fungus, and they shed new light on the forces that have shaped *Coccidioides* genomes.

Our identification of secondary-metabolite clusters expressed in distinct phases of the *C. posadasii* life cycle is consistent with the suites of hyphal- and spherule-unique small molecules observed in this system [46]. Hyphal-specific metabolites may well mediate antagonistic relationships between *Coccidioides* and bacteria in soils [6,47,48]. Likewise, it is tempting to speculate that spherule-specific metabolites govern *C. posadasii* interactions with mammalian hosts, as is the case in other fungal pathogens [49]. As functions become clear for these small molecules, they will likely prove to be critical to the fitness of the organism, given signatures of positive selection between *Coccidioides* species at secondary-metabolite genes [7].

Our discovery of spherule-gene islands in *C. posadasii* mirrors analyses of linked genes co-regulated in infection conditions in other fungal pathogens [50,51]. Are the loci we study here virulence islands? This term is conventionally used for stretches of genome that underlie virulence and are passed around within and between species by horizontal gene transfer, as seen in bacteria [52] and other fungi [53,54,55]. The chromosome III locus, whose presence–absence variation we have traced represents a prime candidate for such a case. Furthermore, its high population differentiation strongly suggests that natural selection in distinct niches drove large-scale gains and losses at this locus. As such, our characterization of the chromosome III region opens a first window onto the principle that virulence determinants matter for fitness in *C. posadasii.* By analogy with other fungal pathogens [17,18,19,20,21,55], *Coccidioides* would tune virulence loci to avoid fitness costs of virulence programs during growth in the environment and/or recognition by hosts during infection. If so, the latter would likely manifest in the small desert mammals thought to be the primary animal vector of the fungus [56,57].

That said, apart from our focal chromosome III site, our population analyses did not reveal gains and losses of other spherule genes, or spherule-gene islands, between *C. posadasii* strain genomes. Many may not lie in rapidly evolving chromosomal domains of the kind established in other fungi (i.e., under the two-speed genome model; [58]). For example, chromosome ends, which are sinks for transposons [5] and genes unique to the genus [7] in *Coccidioides*, show no overall enrichment for spherule genes or islands as we identify them here. We do expect, however, that population sampling of additional strains could ultimately reveal copy number variation at more candidate virulence loci in *C. posadasii*.

Alongside our analyses of presence–absence variation, we also used single-nucleotide variants to detect signatures of rapid evolution of spherule-expressed genes. We also identified hundreds of genes with a preponderance of amino acid changes across strains. On the basis of the low population frequencies of the latter, we suggest an interpretation in which some genes harbor an excess of variants in our analyzed genomes as a product of virulence function and ascertainment bias. This model is contingent on the fact that almost all strains sequenced in the current literature were collected from patient samples (although see [8,59]). Thus, we envision that a rare allele could arise in a given *C. posadasii* population that confers no particular advantage when the fungus is living in the soil, but promotes opportunistic virulence in humans [20]. Such sites would show up at elevated frequency in clinical genomes, reflecting their importance for virulence, but without positive selection, per se, as a driving force. Future work will establish whether and how such loci do, in fact, contribute to infection mechanisms.

In conclusion, our results underscore the power of deep genomic resources that, when fed into analysis methods focused on gene function and evolution, can inform models of *C. posadasii* biology. We expect that such in silico approaches will continue to complement experimental strategies to accelerate progress in the field, elucidating how *Coccidioides* persists in the wild and how it causes disease.

### 4.1. Supplementary Note

In our analysis of the region of *C. posadasii* chromosome III with signal of extensive loss and rearrangement across strains (Figure 4 and Supplementary Table S6), we traced evidence for paralogous relationships between genes of the locus using sequence similarity searches. The complete set of results is schematized in Figure 5; we provide details of the inferred paralogous pairs here. The data revealed three likely duplication events: one involving two contiguous genes induced in spherules (D8B26_005352 to D8B26_005353 paralogous to D8B26_005401 to D8B26_005403, indicated by the orange star in Figure 5); one involving a seven-gene contiguous span with no evidence for spherule-specific expression (D8B26_005372 to D8B26_005378 paralogous to D8B26_005394 to D8B26_005400, indicated by the blue star in Figure 5); and one involving a single additional spherule-induced gene (D8B26_005351 paralogous to D8B26_005411, indicated by the purple star in Figure 5).

## Figures and Tables

**Table 1:** Functional annotation of genes with life cycle-specific expression. Each row reports the results of tests for enrichment of *C. posadasii* genes of the indicated annotation class among those induced in spherules (first two columns) or hyphae (last two columns). *N:* number of genes in category; *p*: resampling-based enrichment p-value; antiSMASH: antibiotics and Secondary Metabolite Analysis Shell; SMCOG: antiSMASH Secondary Metabolite Clusters of Orthologous Groups. Bold values signify *p* < 0.05.

	*N,* Spherule-Upregulated Genes	*p,* Spherule-Upregulated Genes	*N,* Hyphae-Upregulated Genes	*p,* Hyphae-Upregulated Genes
**Transmembrane domain-containing genes**	218	0.59	319	**1.42 × 10^−7^**
**Signal peptide-containing genes**	67	0.27	130	**3.95 × 10^−16^**
**Carbohydrate active enzymes**	32	0.0519	42	**0.0009**
**Genes in antiSMASH secondary metabolite clusters**	63	**0.00016**	53	0.15
**SMCOG annotated genes**	30	**6.15 × 10^−7^**	20	**0.049**

**Table 2:** Genes induced in spherules evolve quickly. Each row reports results from one population-genomic metric of single-nucleotide variation, as analyzed in genes across *C. posadasii* strains: π, average number of nucleotide differences between strain pairs in the Arizona population; D_XY_, absolute divergence between the Arizona and Texas/Mexico/South America populations; P_N_/P_S_, count of non-synonymous variants across the Arizona population relative to that of synonymous variants. The first column reports the median of the indicated metric across all genes. The second, third, and fourth columns report, respectively, the number of genes induced in spherules with sufficient sequence data to calculate each metric, the median of the metric across genes in this gene set, and the results of a resampling-based test for enrichment of high values of the metric in this gene set; the last three columns report the analogous quantities for genes annotated with the phosphotransferase enzyme family. Bold values signify *p* < 0.05.

		Spherule-Upregulated Genes			Phosphotransferase Enzyme Family		
	Genomic Median	*N*	Median	*p*	*N*	Median	*p*
**π**	0.000935	971	0.00106	**3.0 × 10^−7^**	27	0.00176	**0.0001**
***D_XY_***	0.00105	952	0.00115	**0.0002**	27	0.00199	**9.0 × 10^−5^**
***P_N_/P_S_***	0.325	748	0.342	**0.0102**	20	0.583	**0.0073**

## Data Availability

All code for this project is available at https://github.com/clairedubin/cocci_popgen accessed on 24 October 2022.

## Supplementary Materials

The following supporting information can be downloaded at: https://www.mdpi.com/article/10.3390/jof8121235/s1. Supplementary Figure S1: Amino acid variants are at low population frequency in highly divergent protein sequences. Supplementary Figure S2: Copy number variants across the *C. posadasii* genome. Supplementary Figure S3: Gene expression pattern does not predict frequency of copy number variation. Supplementary Figure S4: Transposons in the copy number-variable chromosome III region. Supplementary Figure S5: Chromosome III copy number variation is polyphyletic. Supplementary Table S1: Strains and data sources. Supplementary Table S2: Genes of spherule and hyphae islands. Supplementary Table S3: Population-genomic statistics per gene. Supplementary Table S4: Population-genomic analyses of the chromosome III region. Supplementary Table S5: Atlas and analysis of copy number variants. Supplementary Table S6: Gene annotations in the Chromosome III region.

## References

[1] Crum N.F. (2022). Coccidioidomycosis: A Contemporary Review. Infect. Dis. Ther..

[2] Kollath D.R., Miller K.J., Barker B.M. (2019). The mysterious desert dwellers: *Coccidioides immitis* and *Coccidioides posadasii*, causative fungal agents of coccidioidomycosis. Virulence.

[3] Lewis E.R.G., Bowers J.R., Barker B.M. (2015). Dust Devil: The Life and Times of the Fungus That Causes Valley Fever. PLoS Pathog..

[4] Gorris M.E., Van Dyke M.C.C., Carey A., Hamm P.S., Mead H.L., Uehling J.K. (2021). A Review of *Coccidioides* Research, Outstanding Questions in the Field, and Contributions by Women Scientists. Curr. Clin. Microbiol. Rep..

[5] Kirkland T.N., Fierer J. (2018). *Coccidioides immitis* and posadasii; A review of their biology, genomics, pathogenesis, and host immunity. Virulence.

[6] Chow N.A., Kangiser D., Gade L., McCotter O.Z., Hurst S., Salamone A., Wohrle R., Clifford W., Kim S., Salah Z. (2021). Factors Influencing Distribution of *Coccidioides immitis* in Soil, Washington State, 2016. mSphere.

[7] Sharpton T.J., Stajich J.E., Rounsley S.D., Gardner M.J., Wortman J.R., Jordar V.S., Maiti R., Kodira C.D., Neafsey D.E., Zeng Q. (2009). Comparative genomic analyses of the human fungal pathogens *Coccidioides* and their relatives. Genome Res..

[8] Teixeira M.D.M., Barker B.M., Stajich J.E. (2019). Improved Reference Genome Sequence of *Coccidioides* immitis Strain WA_211, Isolated in Washington State. Microbiol. Resour. Announc..

[9] Teixeira M.D.M., Stajich J.E., Sahl J.W., Thompson G.R., Brem R.B., Dubin C.A., Blackmon A.V., Mead H.L., Keim P., Barker B.M. (2022). A chromosomal-level reference genome of the widely utilized *Coccidioides posadasii* laboratory strain “Silveira”. G3 Genes Genomes Genet..

[10] Teixeira M.M., Alvarado P., Roe C.C., Thompson G.R., Patané J.S.L., Sahl J.W., Keim P., Galgiani J.N., Litvintseva A.P., Matute D.R. (2019). Population Structure and Genetic Diversity among Isolates of *Coccidioides posadasii* in Venezuela and Surrounding Regions. mBio.

[11] Carlin A., Beyhan S., Peña J., Stajich J., Viriyakosol S., Fierer J., Kirkland T. (2021). Transcriptional Analysis of *Coccidioides immitis* Mycelia and Spherules by RNA Sequencing. J. Fungi.

[12] Mandel M.A., Beyhan S., Voorhies M., Shubitz L.F., Galgiani J.N., Orbach M.J., Sil A. (2022). The WOPR family protein Ryp1 is a key regulator of gene expression, development, and virulence in the thermally dimorphic fungal pathogen *Coccidioides posadasii*. PLoS Pathog..

[13] Mead H.L., Roe C.C., Keppler E.A.H., Van Dyke M.C.C., Laux K.L., Funke A., Miller K.J., Bean H.D., Sahl J.W., Barker B.M. (2020). Defining Critical Genes During Spherule Remodeling and Endospore Development in the Fungal Pathogen, *Coccidioides posadasii*. Front. Genet..

[14] Whiston E., Wise H.Z., Sharpton T., Jui G., Cole G.T., Taylor J.W. (2012). Comparative Transcriptomics of the Saprobic and Parasitic Growth Phases in *Coccidioides* spp.. PLoS ONE.

[15] Goranov A.I., Madhani H.D. (2014). Functional Profiling of Human Fungal Pathogen Genomes. Cold Spring Harb. Perspect. Med..

[16] Motaung T.E., Saitoh H., Tsilo T.J. (2017). Large-scale molecular genetic analysis in plant-pathogenic fungi: A decade of genome-wide functional analysis. Mol. Plant Pathol..

[17] Dutheil J.Y., Mannhaupt G., Schweizer G., Sieber C.M., Münsterkötter M., Güldener U., Schirawski J., Kahmann R. (2016). A Tale of Genome Compartmentalization: The Evolution of Virulence Clusters in Smut Fungi. Genome Biol. Evol..

[18] Faino L., Seidl M.F., Shi-Kunne X., Pauper M., van den Berg G.C., Wittenberg A.H., Thomma B.P. (2016). Transposons passively and actively contribute to evolution of the two-speed genome of a fungal pathogen. Genome Res..

[19] Plissonneau C., Stürchler A., Croll D. (2016). The Evolution of Orphan Regions in Genomes of a Fungal Pathogen of Wheat. mBio.

[20] Rokas A. (2022). Evolution of the human pathogenic lifestyle in fungi. Nat. Microbiol..

[21] Siscar-Lewin S., Hube B., Brunke S. (2022). Emergence and evolution of virulence in human pathogenic fungi. Trends Microbiol..

[22] Kirkland T.N., Muszewska A., Stajich J.E. (2018). Analysis of Transposable Elements in *Coccidioides* Species. J. Fungi.

[23] Smit A., Hubley R., Green P. (2013). RepeatMasker Open-4.0. http://www.repeatmasker.org.

[24] Camacho C., Coulouris G., Avagyan V., Ma N., Papadopoulos J., Bealer K., Madden T.L. (2009). BLAST+: Architecture and applications. BMC Bioinform..

[25] Engelthaler D.M., Roe C.C., Hepp C.M., Teixeira M., Driebe E.M., Schupp J.M., Gade L., Waddell V., Komatsu K., Arathoon E. (2016). Local Population Structure and Patterns of Western Hemisphere Dispersal for *Coccidioides* spp., the Fungal Cause of Valley Fever. mBio.

[26] Li H. (2013). Aligning sequence reads, clone sequences and assembly contigs with BWA-MEM. arXiv.

[27] Walker B.J., Abeel T., Shea T., Priest M., Abouelliel A., Sakthikumar S., Cuomo C.A., Zeng Q., Wortman J., Young S.K. (2014). Pilon: An Integrated Tool for Comprehensive Microbial Variant Detection and Genome Assembly Improvement. PLoS ONE.

[28] Danecek P., Bonfield J.K., Liddle J., Marshall J., Ohan V., Pollard M.O., Whitwham A., Keane T., McCarthy S.A., Davies R.M. (2021). Twelve years of SAMtools and BCFtools. GigaScience.

[29] Danecek P., Auton A., Abecasis G., Albers C.A., Banks E., DePristo M.A., Handsaker R.E., Lunter G., Marth G.T., Sherry S.T. (2011). The variant call format and VCFtools. Bioinformatics.

[30] Edgar R.C. (2004). MUSCLE: Multiple sequence alignment with high accuracy and high throughput. Nucleic Acids Res..

[31] Suyama M., Torrents D., Bork P. (2006). PAL2NAL: Robust conversion of protein sequence alignments into the corresponding codon alignments. Nucleic Acids Res..

[32] Sukumaran J., Holder M.T. (2010). DendroPy: A Python library for phylogenetic computing. Bioinform. Oxf. Engl..

[33] De Mita S., Siol M. (2012). EggLib: Processing, analysis and simulation tools for population genetics and genomics. BMC Genet..

[34] Boeva V., Popova T., Bleakley K., Chiche P., Cappo J., Schleiermacher G., Janoueix-Lerosey I., Delattre O., Barillot E. (2012). Control-FREEC: A tool for assessing copy number and allelic content using next-generation sequencing data. Bioinform. Oxf. Engl..

[35] Steenwyk J.L., Soghigian J.S., Perfect J.R., Gibbons J.G. (2016). Copy number variation contributes to cryptic genetic variation in outbreak lineages of *Cryptococcus gattii* from the North American Pacific Northwest. BMC Genom..

[36] Haridas S., Albert R., Binder M., Bloem J., LaButti K., Salamov A., Andreopoulos B., Baker S., Barry K., Bills G. (2020). 101 Dothideomycetes genomes: A test case for predicting lifestyles and emergence of pathogens. Stud. Mycol..

[37] Shi-Kunne X., Faino L., Berg G.C.M.V.D., Thomma B.P.H.J., Seidl M.F. (2017). Evolution within the fungal genus Verticillium is characterized by chromosomal rearrangement and gene loss. Environ. Microbiol..

[38] Redon R., Ishikawa S., Fitch K.R., Feuk L., Perry G.H., Andrews T.D., Fiegler H., Shapero M.H., Carson A.R., Chen W. (2006). Global variation in copy number in the human genome. Nature.

[39] Fisher M.C., Koenig G.L., White T.J., San-Blas G., Negroni R., Alvarez I.G., Wanke B., Taylor J.W. (2001). Biogeographic range expansion into South America by *Coccidioides immitis* mirrors New World patterns of human migration. Proc. Natl. Acad. Sci. USA.

[40] Moxon R., Bayliss C., Hood D. (2006). Bacterial Contingency Loci: The Role of Simple Sequence DNA Repeats in Bacterial Adaptation. Annu. Rev. Genet..

[41] Jamie G.A., Meier J.I. (2020). The Persistence of Polymorphisms across Species Radiations. Trends Ecol. Evol..

[42] Llaurens V., Whibley A., Joron M. (2017). Genetic architecture and balancing selection: The life and death of differentiated variants. Mol. Ecol..

[43] Qi X., Li X., Guo H., Guo N., Cheng H. (2018). *Vd*PLP, A Patatin-Like Phospholipase in *Verticillium dahliae*, Is Involved in Cell Wall Integrity and Required for Pathogenicity. Genes.

[44] Nayak A.P., Green B.J., Beezhold D. (2013). Fungal hemolysins. Med Mycol..

[45] Theiss S., Ishdorj G., Brenot A., Kretschmar M., Lan C.-Y., Nichterlein T., Hacker J., Nigam S., Agabian N., Köhler G.A. (2006). Inactivation of the phospholipase B gene PLB5 in wild-type Candida albicans reduces cell-associated phospholipase A2 activity and attenuates virulence. Int. J. Med Microbiol..

[46] Keppler E.A.H., Mead H.L., Barker B.M., Bean H.D. (2021). Life Cycle Dominates the Volatilome Character of Dimorphic Fungus *Coccidioides* spp.. mSphere.

[47] Egeberg R.O., Elconin A.E., Egeberg M.C. (1964). Effect of salinity and temperature on *Coccidioides immitis* and three antagonistic soil saprophytes. J. Bacteriol..

[48] Lauer A., Baal J.D., Mendes S.D., Casimiro K.N., Passaglia A.K., Valenzuela A.H., Guibert G. (2019). Valley Fever on the Rise—Searching for Microbial Antagonists to the Fungal Pathogen *Coccidioides immitis*. Microorganisms.

[49] Kuhnert E., Collemare J. (2022). A genomic journey in the secondary metabolite diversity of fungal plant and insect pathogens: From functional to population genomics. Curr. Opin. Microbiol..

[50] Cairns T., Minuzzi F., Bignell E. (2010). The host-infecting fungal transcriptome. FEMS Microbiol. Lett..

[51] Kellner R., Bhattacharyya A., Poppe S., Hsu T., Brem R.B., Stukenbrock E.H. (2014). Expression Profiling of the Wheat Pathogen Zymoseptoria tritici Reveals Genomic Patterns of Transcription and Host-Specific Regulatory Programs. Genome Biol. Evol..

[52] Gal-Mor O., Finlay B.B. (2006). Pathogenicity islands: A molecular toolbox for bacterial virulence. Cell. Microbiol..

[53] Lind A.L., Wisecaver J.H., Lameiras C., Wiemann P., Palmer J.M., Keller N.P., Rodrigues F., Goldman G.H., Rokas A. (2017). Drivers of genetic diversity in secondary metabolic gene clusters within a fungal species. PLoS Biol..

[54] Sacristán S., Goss E.M., Akker S.E.-V.D. (2021). How do pathogens evolve novel virulence activities?. Mol. Plant-Microbe Interact..

[55] Zhao S., Gibbons J.G. (2018). A population genomic characterization of copy number variation in the opportunistic fungal pathogen *Aspergillus fumigatus*. PLoS ONE.

[56] Reyes-Montes M.D.R., Pérez-Huitrón M.A., Ocaña-Monroy J.L., Frías-De-León M.G., Martínez-Herrera E., Arenas R., Duarte-Escalante E. (2016). The habitat of *Coccidioides* spp. and the role of animals as reservoirs and disseminators in nature. BMC Infect. Dis..

[57] Taylor J.W., Barker B. (2019). The endozoan, small-mammal reservoir hypothesis and the life cycle of *Coccidioides* species. Med. Mycol..

[58] Dong S., Raffaele S., Kamoun S. (2015). The two-speed genomes of filamentous pathogens: Waltz with plants. Curr. Opin. Genet. Dev..

[59] Litvintseva A.P., Marsden-Haug N., Hurst S., Hill H., Gade L., Driebe E.M., Ralston C., Roe C., Barker B., Goldoft M. (2014). Valley Fever: Finding New Places for an Old Disease: *Coccidioides immitis* Found in Washington State Soil Associated with Recent Human Infection. Clin. Infect. Dis. Off. Publ. Infect. Dis. Soc. Am..

