# Supplementary material for: Genome Organization and Copy-Number Variation Reveal Clues to Virulence Evolution in Coccidioides posadasii
Source: J Fungi (Basel). 2022 Nov 22;8(12):1235. doi: 10.3390/jof8121235 (PMC9782707; doi:10.3390/jof8121235)
Supplement: Supplementary file 1 [file jof-08-01235-s001.zip › jof-2020636-supplementary/Dubin_Supplementary_Files/Dubin_supplementary_figures.pdf]

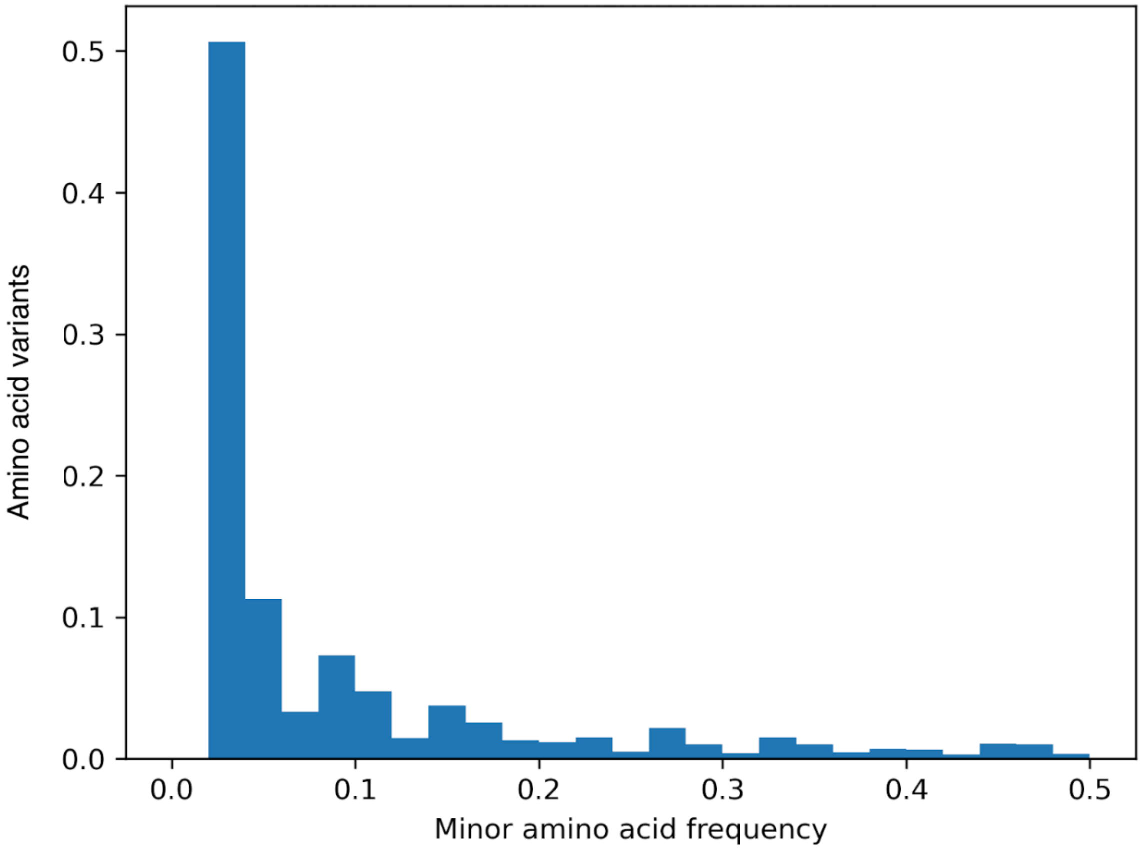

Figure S1. Amino acid variants are at low population frequency in highly divergent protein sequences. Shown are the results of allele frequency analysis of genes in which amino acid variants across Arizona *C. posadasii* strains, called relative to the Silveira reference genome, were in excess relative to synonymous changes (Table S1). The *x*-axis reports the frequency of the non-reference allele of a given amino acid variant, and the y-axis reports the proportion of variants with the frequency on the *x*.

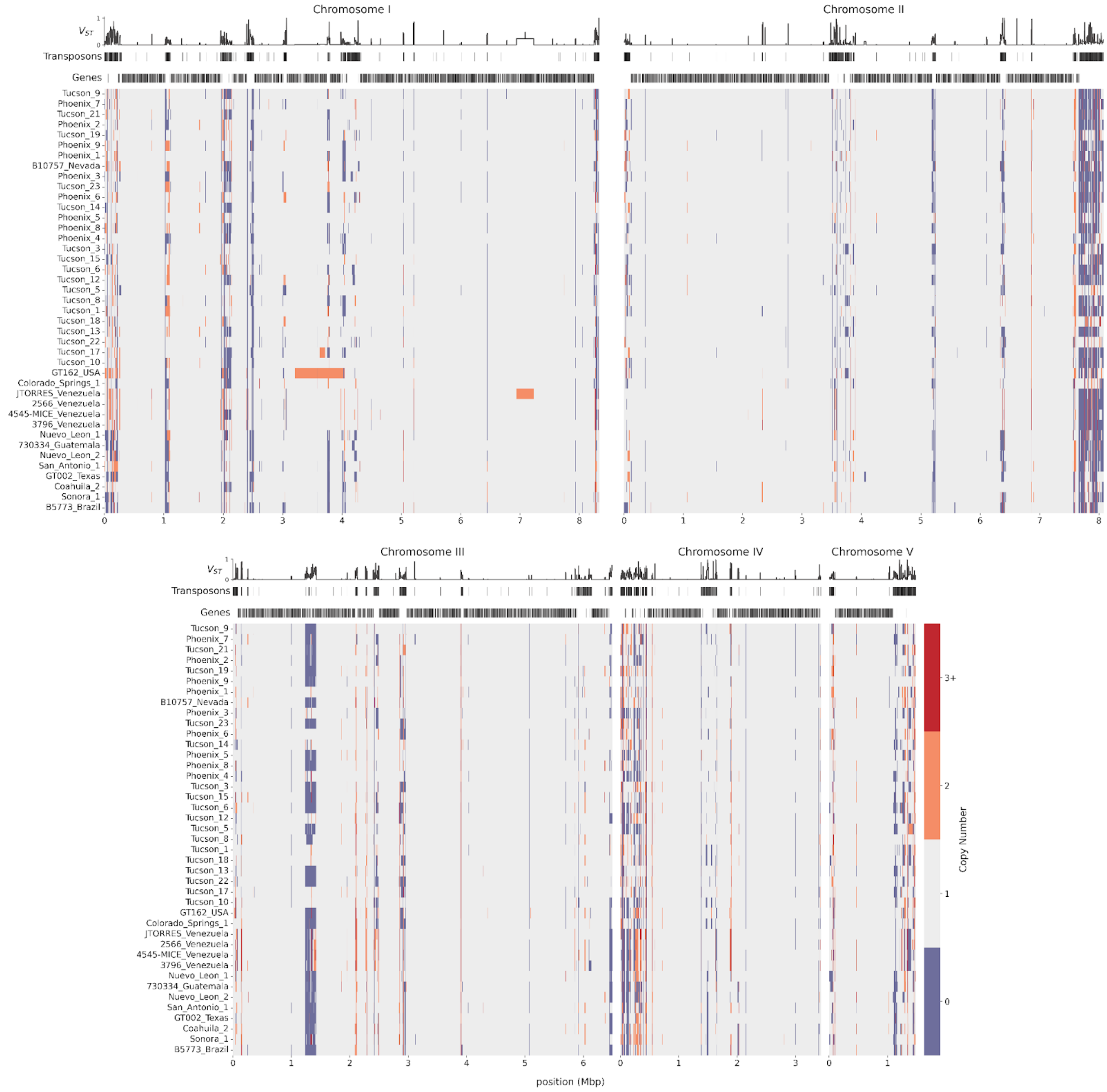

Figure S2. Copy number variants across the *C. posadasii* genome. Data are as in Figure 3 of the main text except that all chromosomes are shown.

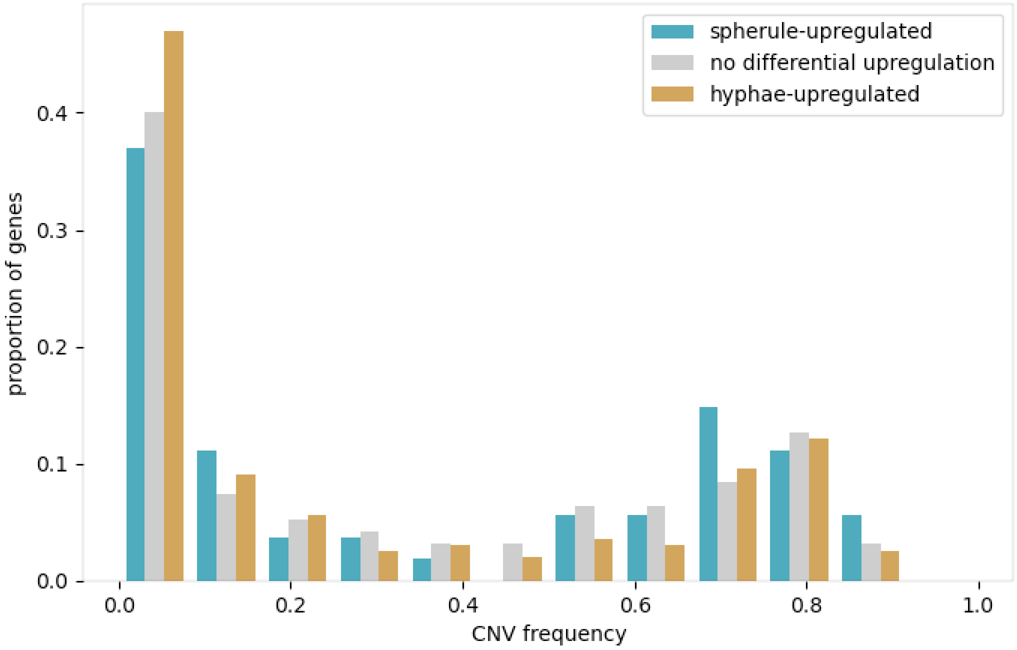

Figure S3. Gene expression pattern does not predict frequency of copy number variation. For each gene falling into a genomic locus exhibiting copy number variation (CNV) across Arizona *C. posadasii* strains, the frequency of the non-reference allele was tabulated. Each color reports the distribution of such frequencies for genes with the indicated expression pattern: the x-axis reports the frequency and the y-axis reports the proportion of genes with the frequency on the x.

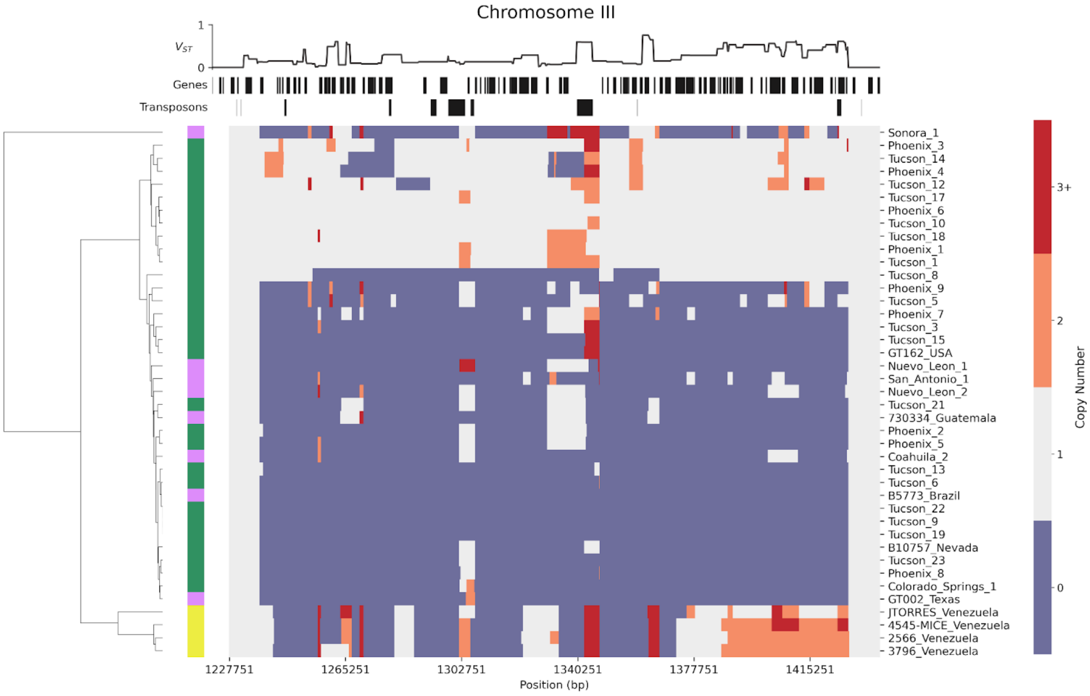

Figure S4. Transposons in the copy number-variable chromosome III region. Data are as in Figure 4 of the main text except that transposons are included, denoted as in Figure 3 of the main text.

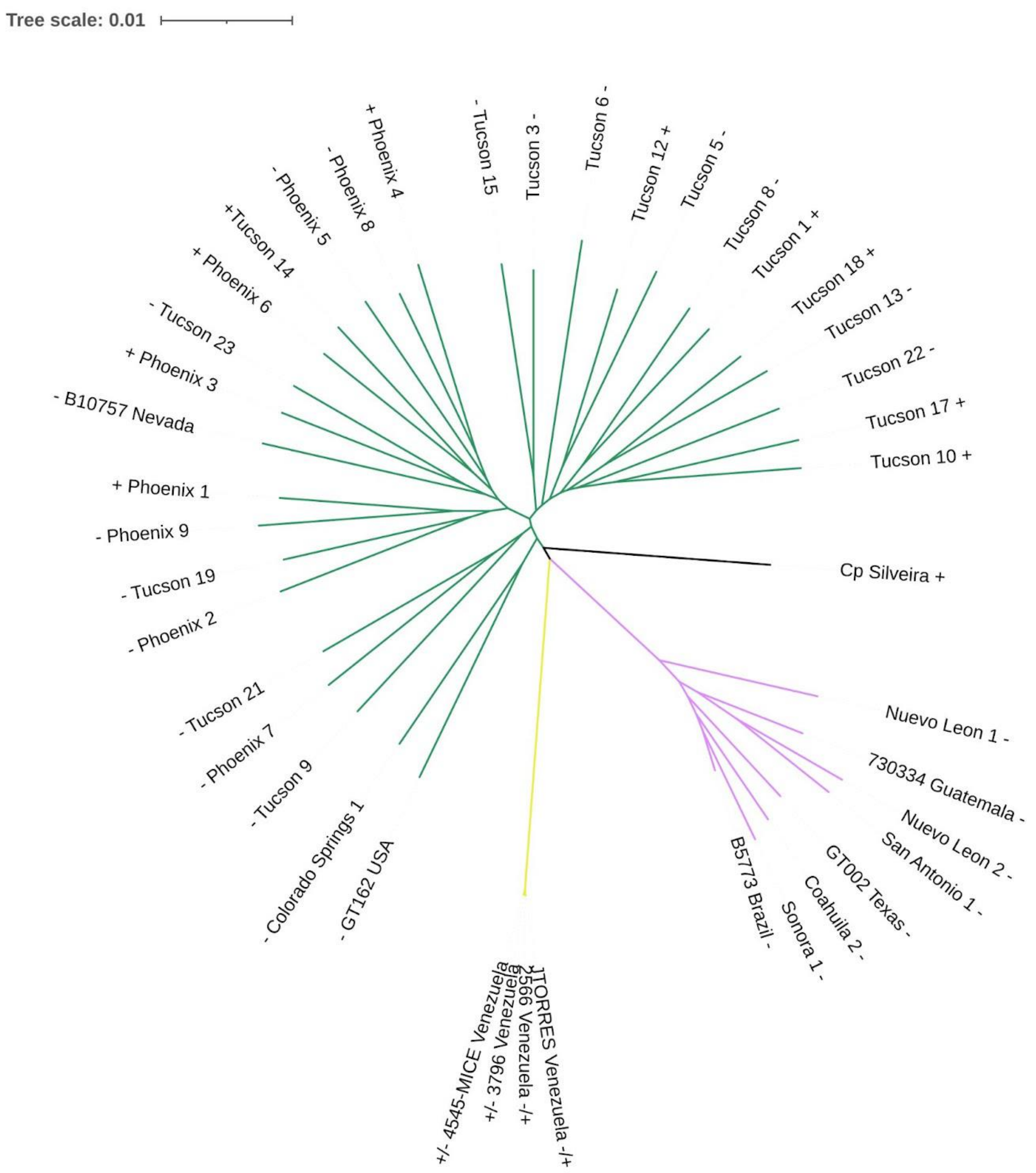

Figure S5. Chromosome III copy number variation is polyphyletic. Shown is the phylogeny of *C. posadasii* strains from (Teixeira et al., 2021): Arizona population, green; Caribbean, yellow; Texas/Mexico/South America, pink. + and – indicate that the respective strain harbors or lacks a version of the chromosome III locus with content as in the Silveira reference genome.
