# Supplementary material for: Genome Organization and Copy-Number Variation Reveal Clues to Virulence Evolution in Coccidioides posadasii
Source: J Fungi (Basel). 2022 Nov 22;8(12):1235. doi: 10.3390/jof8121235 (PMC9782707; doi:10.3390/jof8121235)
Supplement: Supplementary file 1 [file jof-08-01235-s001.zip › jof-2020636-supplementary/Dubin_Supplementary_Files/Dubin_Supplementary_Table_captions.pdf]

Table S1. Each row reports the results of analyses of single nucleotide variation in the indicated gene. The first and second columns report nucleotide diversity (π) within the AZ and TX/MX/SA populations, respectively. The third column reports nucleotide diversity (D_{XY}) between the AZ and TX/MX/SA populations; the fourth column reports the amino acid substitution rate (P_N/P_S) within the AZ population. NaN indicates that a calculation could not be made due to insufficient population data or division by zero errors.

Table S2. In a given tab, each row reports characteristics of a gene falling into a spherule-gene island or hyphal-gene island as defined in Figure 2A or 2B, respectively. The first column reports a window identifier corresponding to the genomic position from left to right in Figure 2. The second through sixth columns report genomic location and GenBank annotation (Teixeira et al., 2021), and the seventh column reports expression data (Mandel et al., 2022).

Table S3. (A) Each row reports the characteristics of a copy number variant called in the indicated strain. The first, second, and third columns report genomic location; the fourth row reports inferred copy number relative to the Silveira *C. posadasii* reference genome. (B) Each row reports the evidence for copy number variation in the indicated 250bp window in the genome. The third column reports population differentiation (V_{ST}) between the Arizona, Texas/Mexico/South America, and Caribbean populations; the fourth column reports any genes overlapping with the indicated window; all remaining columns report inferred copy number in the indicated strains. NaNs in the V_{ST} column indicate windows where all strains have a copy number of 1 and V_{ST} cannot be calculated.

Table S4. Each row reports characteristics of a gene falling into the copy-number-variable locus on chromosome III shown in Figure 4 of the main text. The second column reports expression patterns from (Mandel et al., 2022); the third column reports annotation data from GenBank (Teixeira et al., 2021); the fourth and fifth columns report inferred *C. immitis* homologs and *Uncinocarpus reesii* homologs, respectively. The last column reports inferred paralogs encoded within the locus.

Table S5. Each tab reports results from a comparison of two groups of Arizona strains categorized with respect to the copy-number-variable locus on chromosome III shown in Figure 4 of the main text: strains with the locus inferred to be present in the genome and those with an inferred absence of the locus. (A) Each row reports the results of analyses of single-nucleotide variation in the indicated 250bp genomic window. The third and fourth columns report nucleotide diversity (π) of the window among strains with and without the chromosome III locus, respectively; the fifth and sixth columns report Tajima's D among strains with and without the locus, respectively. The last column reports population differentiation (F_{ST}) between strains with and without the locus. NaN indicates that a value could not be calculated because no variants exist in the region. (B) Each set of rows reports the results of tests for enrichment, in sequence up- or downstream respectively of the chromosome III locus, of extreme values of metrics of single-nucleotide variation between strains with and without the locus. In the first two rows with data, value_tested reports nucleotide diversity across strains with and without the chromosome III locus respectively, in each case as a maximum among windows within 5000 bp upstream of the chromosome III locus 5' end; p_value reports the empirical resampling-based *p* value of this maximum π value, against a genomic null. The fourth and fifth rows with data report analogous calculations using Tajima's D as the focal statistic. The third row with data reports analyses of F_{ST} between strains with and without the locus, as a maximum among windows upstream of the chromosome III locus. The bottom five rows report analogous calculations among windows within 5000 bp downstream of the chromosome III locus 3' end.

Table S6. Each row reports the SRA accession number, original publication, and country of origin for a given strain in this study. Population classifications and admixture calculations are from (Teixeira et al., 2019); each strain was included in population-level calculations in this study only if Admixed=False. The “Concordant strains” column lists strains from Teixeira et al., 2019 that were not included in our analysis due to high genome concordance with the strain listed at the start of the row. The "Year Isolated" column lists the year the sample was isolated according to (Engelthaler et al. 2016) or NA if the information is not available.
